# Surgical repair in case of covered exstrophy of bladder with complete duplication of lower genitourinary tract and visceral sequestration

**DOI:** 10.1590/S1677-5538.IBJU.2016.0634

**Published:** 2018

**Authors:** Sachin Sarode, Sunil Mhaske, Vinayak G. Wagaskar, Bhushan Patil, Sujata K. Patwardhan, Ganesh Gopalakrishnan

**Affiliations:** 1 Department of Urology, Seth GSMC and King's Edward Memorial Hospital, Mumbai, India; 2 Department of Urology, Dr. D. Y. Patil Medical College Pimpri, Pune, India; 3 Department of Urology, Vednayagam Hospital, Coimbatore, India

## PURPOSE

Management of complete lower urinary tract duplication remains a major challenge. We present a video-case of covered exstrophy of bladder with complete duplication of lower genitourinary tract and visceral sequestration.

## MATERIALS AND METHODS

An eleven year old female child presented with abnormal structure over the genital area since birth and continuous urine leak. Patient had history of premature birth at 28 weeks of gestation. Anus was anteriorly displaced in exstrophy complex. Labia majora and minora were divergent and clitoris was bifid. Two normal vaginas were present on both sides lateral to genito- urinary complex structure. Patient had single urethral meatus which opened above right vaginal orifice.

MRI pelvis was suggestive of pubic diastasis with duplication of urinary bladder, uterine didelphys with two ovaries and two vaginas. Excretory urography confirmed duplication of urinary bladder with opening of right ureter into right bladder and left ureter into left bladder. Patient had left sided hydro-ureteronephrosis and no demonstrable outlet. There was herniation of right urinary bladder through anterior abdominal wall defect and urethra was originating only from right bladder.

Surgical procedure consisted of joining of both the bladders and dissection of urethra. Decision to excise the urethra was taken as there was no demonstrable bladder neck sphincter; Mitrofanoff procedure was done for drainage. External genitalia reconstruction consisted of clitoroplasty, mons reconstruction and labioplasty. Sequestered part was excised; pubic bone defect was closed with raising rectus sheath flap.

## CONCLUSION

Comprehensive preoperative imaging and meticulous planning is needed for management of rare and complicated lower urogenital anomalies. Surgical procedure achieved all of the preoperative goals in this case. Quality of life improved with cessation of continuous dribbling and improved cosmesis of external genitalia. Functional outcome improved with drainage of left side urinary system into the unified lower urinary tract.

## ARTICLE INFO

**Available at: http://www.intbrazjurol.com.br/video-section/20160634_Sarode_et_al Int Braz J Urol. 2018; 44 (Video #9): 647-8**

